# Clinical course and prognosis of musculoskeletal pain in patients referred for physiotherapy: does pain site matter?

**DOI:** 10.1186/s12891-017-1487-3

**Published:** 2017-03-29

**Authors:** Nils-Bo de Vos Andersen, Peter Kent, Jakob Hjort, David Høyrup Christiansen

**Affiliations:** 1 Primary Health Care and Quality Improvement, Viborg, Central Denmark Region Denmark; 2 School of Physiotherapy and Exercise Science, Curtin University, Bentley, WA Australia; 3 Department of Sports Science and Clinical Biomechanics, University of Southern Denmark, Odense, Denmark; 4 Department of Clinical Medicine, HEALTH, Aarhus University, Aarhus, Denmark; 5 Department of Occupational Medicine, Regional Hospital West Jutland–University Research Clinic, Herning, Denmark

**Keywords:** Musculoskeletal pain, Physiotherapy, Cohort study, Prognosis

## Abstract

**Background:**

Danish patients with musculoskeletal disorders are commonly referred for primary care physiotherapy treatment but little is known about their general health status, pain diagnoses, clinical course and prognosis.

The objectives of this study were to 1) describe the clinical course of patients with musculoskeletal disorders referred to physiotherapy, 2) identify predictors associated with a satisfactory outcome, and 3) determine the influence of the primary pain site diagnosis relative to those predictors.

**Methods:**

This was a prospective cohort study of patients (*n* = 2,706) newly referred because of musculoskeletal pain to 30 physiotherapy practices from January 2012 to May 2012. Data were collected via a web-based questionnaire 1–2 days prior to the first physiotherapy consultation and at 6 weeks, 3 and 6 months, from clinical records (including primary musculoskeletal symptom diagnosis based on the ICPC-2 classification system), and from national registry data. The main outcome was the Patient Acceptable Symptom State. Potential predictors were analysed using backwards step-wise selection during longitudinal Generalised Estimating Equation regression modelling. To assess the influence of pain site on these associations, primary pain site diagnosis was added to the model.

**Results:**

Of the patients included, 66% were female and the mean age was 48 (SD 15). The percentage of patients reporting their symptoms as acceptable was 32% at 6 weeks, 43% at 3 months and 52% at 6 months. A higher probability of satisfactory outcome was associated with place of residence, being retired, no compensation claim, less frequent pain, shorter duration of pain, lower levels of disability and fear avoidance, better mental health and being a non-smoker. Primary pain site diagnosis had little influence on these associations, and was not predictive of a satisfactory outcome.

**Conclusion:**

Only half of the patients rated their symptoms as acceptable at 6 months. Although satisfactory outcome was difficult to predict at an individual patient level, there were a number of prognostic factors that were associated with this outcome. These factors should be considered when developing generic prediction tools to assess the probability of satisfactory outcome in musculoskeletal physiotherapy patients, because the site of pain did not affect that prognostic association.

**Electronic supplementary material:**

The online version of this article (doi:10.1186/s12891-017-1487-3) contains supplementary material, which is available to authorized users.

## Background

Musculoskeletal disorders are a common cause of ill-health, with substantial personal, community and societal consequences [1, 2]. In Denmark, over 50% of the adult population will have experienced pain or discomfort within the last 14 days, and 37% report that they have been severely bothered by their pain [3]. Musculoskeletal pain is one of the most common reasons that people seek medical help in primary care, with up to 30% having at least one contact with their general practitioner due to musculoskeletal pain conditions over a period of 18 months [4]. In Denmark, patients with musculoskeletal pain are often referred for physiotherapy treatment [5], but the general health status, distribution of pain diagnoses and clinical course in these patients has not been described.

Identifying prognostic factors and subgroups of patients who respond best to physiotherapy interventions is considered an important research priority [6, 7]. Therefore, in the last two decades a large number of studies have identified potential predictors of outcomes in different musculoskeletal pain conditions [8–12]. Predictive factors can vary depending on differences in study populations, settings, statistical procedures and outcome measures [13].

Commonly used outcome measures in musculoskeletal pain prognostic studies have been improvement in pain, improvement in function or various definitions of ‘disabling pain’, but there are reasons why outcome measures that tap into broader aspects of recovery might be suitable alternative measures of outcome. The first reason is that the relationship between pain and function is strongly influenced by psychosocial attributes, such as coping, catastrophisation, pain beliefs and pain self-efficacy [14]. Secondly, because of the fluctuating clinical course of much musculoskeletal pain, the relatively low likelihood of complete recovery, and the high recurrence rate [15], contemporary treatment includes a focus on promoting active coping strategies despite pain and on restoring functional activity [16], and therefore outcome measures ideally would tap into these constructs. Therefore, outcome measures that assess the acceptability of a symptom state not only assess patients’ adaption to their health condition but also are not restricted to a single dimension of their condition.

The Patient Acceptable Symptom State (PASS) is measured using a question that assesses the level of symptoms beyond which patients consider themselves well and, while it was originally developed for patients with rheumatic disorders [17, 18], it may also be appropriate for more broad use in musculoskeletal pain conditions. To our knowledge, the PASS has not been previously applied as an indicator of a satisfactory outcome in patients with diverse musculoskeletal disorders referred to primary care physiotherapy and the predictors associated with this outcome have not been previously evaluated in this context.

In addition, most prognostic factor research in musculoskeletal pain has been centred on people with pain in specific anatomical pain sites (e.g. low back pain, neck pain or shoulder pain) [8–10]. However, there is a view that some prognostic factors are likely to be similar, regardless of the specific pain site. For example, a recent systematic review that compared primary care prognostic factors across different anatomical pain sites, found that despite considerable heterogeneity between studies, some factors consistently emerged across different regional pain complaints [19]. As clinicians in primary care often treat a variety of musculoskeletal pain patients, such knowledge is useful. These findings also suggest that, as different regional pain syndromes share similar prognostic attributes, in musculoskeletal pain these may be more prognostic than the primary pain site diagnosis itself [6].

Therefore, the three objectives of the study were to: 1) describe the clinical management and course of Danish patients referred to physiotherapy due to musculoskeletal pain, on the outcomes of pain intensity, disability (activity limitation), sick leave and whether they perceived their symptoms as acceptable at follow-up (satisfactory outcome), 2) identify predictors associated with satisfactory outcome, and 3) investigate the influence of primary pain site diagnosis on the strength of those associations.

## Methods

### Study design and population

This was a prospective cohort study conducted in primary-care physiotherapy practices in Denmark. Denmark is divided into five geographical regions, with each region administering its own public hospitals and primary health care services, including physiotherapy practices. A total of 30 physiotherapy practices in four regions (Capital Region of Denmark, Region of Southern Denmark, Central Denmark Region, Northern Denmark Region) participated in the study. Each physiotherapist was requested to collect completed questionnaires and clinical data on newly referred patients with musculoskeletal disorders in the period from January 2012 to May 2012. Consecutive patients were invited to participate if they fulfilled the following inclusion criteria: aged 18 years or above, able to understand Danish well enough to self-complete the questionnaires, not referred for home treatment, and not having received physiotherapy treatment for the same problem in the preceding 3 months. All participants signed written informed consent forms and the study was approved by the Danish Data Protection Agency (No. 2007-58-0010). As treatment was not affected by participation in the study, under Danish law, this study did not need ethics approval *(Act on Research Ethics Review of Health Research Projects, October 2013)* [20].

### Data collection

Questionnaire and clinical data were collected using an existing web-based clinical database (www.fysdb.dk). Patients who agreed to participate in the study were asked to complete on-line questionnaires 1–2 days prior to the first physiotherapy consultation (baseline) and at 6 weeks, 3 and 6 months follow up. Participants were notified by e-mail when the follow-up questionnaires were available for completion. The questionnaires included items about education, weight, height, smoking, and physical activity, as previously used in other Danish population-based surveys [3]. Pain, disability, pain behavior, sleep and mental health were measured by the following validated questionnaires: Standard Evaluation Questionnaire (SEQ) [21–23], the Örebro Musculoskeletal Pain Screening Questionnaire (MSPQ) [24, 25] and the Mental Health Scale – Five (MH-5) [26].

As patients who are referred to physiotherapy have diverse musculoskeletal disorders, we used a multidimensional assessment tool (SEQ) that was not specific to only one body region. The SEQ assesses pain, disability, and sleep disturbance in three separate modules: SEQ-pain [21], SEQ-disability [22] and SEQ-sleep [23]. In contrast to most other musculoskeletal pain assessment tools, it can be used with pain in the upper limbs, lower limbs or spinal regions. As a Danish language version of the SEQ was not available, as part of this study, the SEQ modules were forward and back translated, cross-culturally adapted and its measurement properties evaluated using internationally recommended methods [27–29]. Test-retest reliability of the Danish version of the SEQ was deemed to be acceptable and construct validity was described relative to validated region-specific scales [30–34], with the results being similar to those in the original language version [21, 23]. (See Additional file 1 for more detail). Lastly, information on health-related income support, physiotherapy interventions received and number of consultations was obtained by the Danish National Register on Public Transfer Payments [35] and the National Health Service Register [36],

### Prognostic factors

#### Primary pain site diagnosis

At the initial physiotherapy consultation, the patients’ primary musculoskeletal pain site diagnosis was recorded using the International Classification for Primary Care 2^nd^ edition (ICPC-2) system [37], which is reliable and valid for classifying musculoskeletal disorders [38]. Prior to the start of the study, all participating physiotherapists were invited to attend a 4-h workshop to standardise the method of data collection. Additional descriptions of the data collection procedures were made available to clinicians via the on-line clinical registry software.

#### Potential prognostic variables

Based on previous literature [8, 19, 39], the following candidate prognostic factors from four health domains were included:

1. *Sociodemographic factors* which included *sex, age, educational level* (categorised into unskilled, lower level (<3 years), vocational and training, medium level (3–4 years), higher level (>4 years), and other), g*eographical region* (i.e. the physiotherapy practice’s location in one of four regions), *information on health-related income support received* (classified into none (no record of transfer payments), temporary (sickness benefit, vocational rehabilitation benefit, or cash benefit, except if due to unemployment), permanent (disability pension, or voluntary early retirement that may be due to health reasons), flex-job (which is an income subsidised job due to limited work capacity) or age retirement). These definitions have been used to quantify social and economic consequences of health-related disability in other prospective cohort studies [40, 41]. Furthermore, information on *private health insurance*, any ongoing *compensation claim (litigation)* for their current pain condition, or previous claim was obtained;
2. *Pain and Function* were measured by the SEQ pain and disability modules, which included (i) items on *duration of pain* (<1 month, 1–3 months, 4–12 months and >12 months), *frequency of pain* (constant, daily basis, 2–6 days a week, 1–4 times a month and less often) and *pain medication* (several times a day, once a day, 2–6 days a week, 1–4 times a month, rarely, and never), *pain location and number of pain sites* (shown on a pain chart), *pain intensity* in body regions scored on a numerical rating scale (0 = no pain, 10 = intolerable pain), (ii) a subscale of *pain during activity* consisting of 12 items (0 = no pain, 10 = intolerable pain), and (iii) a subscale of *disability* consisting of 12 items (0 = no difficulties, 10 = not possible to perform) covering activities of daily living in three sections, namely, the upper, lower and spinal body regions, that were converted into a sum score 0–100, where 0 represented no pain/no difficulties;
3. *Psychological factors* included (i) three questions on *fear of physical activity in leisure and work activities*, each scored on a 0 to 10 numeric scale (0 = completely agree, 10 = completely disagree) and added to become a sum score (0–30), (ii) one question on *ability to cope and deal with pain* (0 = no possibility, 10 = completely) from the MSPQ [24, 42], (iii) the SEQ sleep module, which contained two questions on *sleep problems* and *influence of sleep problems in daytime activity* scored on a 0 to10 numeric scale (0 = no problems, 10 = severe problems) converted into a sum score 0–100 [23], and (iv) five questions on *well-being* scored on a 5-point Likert scale from the MH-5 [26] converted into a score 0–100 (0 = worst possible state); and
4. *Health behaviour,* including being a *smoker*, *Body Mass Index* calculated from self-reported height and weight, and *days per week being physical active >30 min* scored on an 8-point scale ranging from 0 = none to 7 = every day.

### Outcome

The main outcome was the Patient Acceptable Symptom State (PASS) measured by a single-item question adapted from the original question designed for rheumatology patients [17, 18], that has also been found useful as an outcome measure for patients with knee and hip osteoarthritis [43]. The wording of the question was ‘*Taking into account the many ways your pain affects your daily life, if you were to remain for the next few months as you are now, would you consider your current state to be satisfactory”* The question was answered by selecting either the ‘yes’ or ‘no’ box. The capacity for the PASS to define the highest level of symptom at which patients find their condition acceptable [44] has been shown to be stable over time [45] and across different patient-reported outcome domains (i.e. pain, function and global assessment) [43].

### Data analysis

Descriptive statistics (percentages, means) were used to report the clinical course. We calculated the number of missing values, follow-up response rates, and differences in baseline characteristics between responders and non-responders at each time point and across administrative regions. For each time point, pain and disability scores, the percentages of patients being on temporary health-related benefits, and the proportion of participants who perceived their symptoms as acceptable (PASS), were calculated and change over time was analysed by longitudinal linear mixed models for continuous variables and Generalised Estimating Equation (GEE) models for categorical variables [46].

Analysis of predictors for satisfactory outcomes was performed by multivariable regression modelling (GEE). Consideration of the number of variables to include in multivariable model building was based on the rule-of-thumb that a least 10 cases per variable were needed to avoid over-fitting the model [47, 48]. Initially, candidate variables were checked for their univariate association with the outcome and for possible collinearity. Where variables were highly correlated (>0.7), only one of the variables was retained. For continuous variables, the log-odds linearity assumption was checked; in case of non-linearity, the variable was recoded as a categorical variable. Next, a stepwise backward regression analysis was performed. At each step, the variable with the highest *p*-value was removed from the model until all remaining variables had a p-value below 0.05. Then the variable primary symptom diagnosis (ICPC-2 classification) was added to the model and the models were compared. To explore if the significance level would affect the inclusion of variables, sensitivity analysis was performed by repeating the stepwise backward regression analysis using a p-value below 0.2 to retain variables. In addition, the possible influence of further sub-grouping symptom diagnosis was explored by reclassifying neck and low back patients into those with or without radiating pain. All statistical analyses were performed using Stata Version 13 (StataCorp LP, College Station, TX, USA).

## Results

### Baseline characteristics

**Fig. 1:**
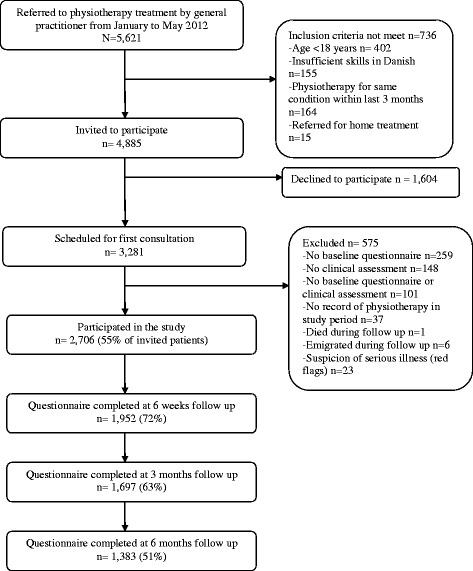
Flow of participants through the study

The flow of participants within the study is shown in Fig. 1. A total of 4,885 patients who were referred to physiotherapy matched the inclusion criteria and 2,706 (55%) consented to participate in the study. Their baseline characteristics are presented in Table 1. Missing values did not exceed 3% for any baseline variable. Patients who failed to complete any follow-up questionnaires were more often men than women, unskilled or with lower educational level, using pain medication, smokers, having more co-morbidity, less physically active and having higher pain, disability and psychological scores (*p* < 0.05).

**Table 1:** Baseline characteristics (*n* = 2,706)

Socio-demographic		
Geographical Regions, n (%)		
Capital Region of Denmark	594	(22.0)
Region of Southern Denmark	735	(27.2)
Central Denmark Region	499	(18.4)
North Denmark Region	878	(32.4)
Female, n (%)	1.798	(66.4)
Age, mean (SD)	48.3	(15.1)
Education, n (%)		
Unskilled	392	(14.8)
Lower level (<3 years)	596	(22.5)
Vocational and training	426	(16.1)
Medium level (3–4 years)	802	(30.3)
Higher level (>4 years)	315	(11.9)
Other	118	(4.5)
Health-related benefits, n (%)		
None	1.776	(65.6)
Temporary	245	(9.1)
Permanent	181	(6.7)
Retired	504	(18.6)
Private health insurance, n (%)	798	(30.1)
Compensation claim (litigation), n (%)	139	(5.2)
Pain and Function		
Pain duration		
< 1 month	640	(24.2)
1–3 months	739	(27.9)
4–12 months	505	(19.1)
> 12 months	766	(28.9)
Pain score 0–10,mean (SD)	6.5	(2.3)
Number of pain sites 0–9, mean (SD)	2.8	(1.7)
Pain during activity score 0–100, mean (SD)	32.7	(20.6)
Disability score 0–100, mean (SD)	26.2	(20.0)
Frequency of pain, n (%)		
Constant	979	(36.9)
Daily basis	1,341	(50.6)
2–6 times a week	212	(8.0)
1–4 times a month	76	(2.9)
Less often	42	(1.6)
Taking pain medication, n (%)		
Several times a day	818	(30.9)
Once a day	243	(9.2)
2–6 times a week	518	(19.5)
1–4 times a month	383	(14.5)
Less often	226	(8.5)
Never	462	(17.4)
Co morbidity, n (%)		
1	591	(22.3)
2 or more	224	(8.5)
Previous episode, n (%)	1.357	(50.7)
Psychological factors		
Fear avoidance 0–30, mean (SD)	15.8	(8.4)
Coping skills 0–10, mean (SD)	5.4	(2.8)
Sleep score 0–100, mean (SD)	35.3	(29.0)
Mental health score 0–100, mean (SD)	75.0	(17.6)
Health behaviour		
Smoker, n (%)	532	(19.7)
Body Mass Index, mean (SD)	26.1	(4.7)
Physical activity ≥30 min.0–7 days, mean (SD)	4.1	(2.5)

*Abbreviation*: *SD* standard deviation

### Primary symptom diagnosis and treatment

**Fig. 2:**
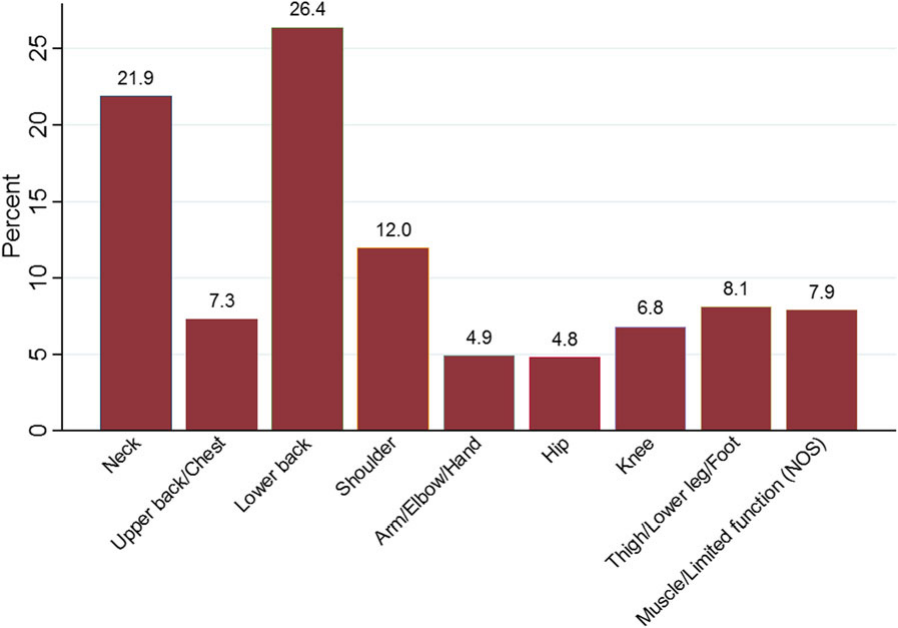
Primary pain diagnosis (ICPC-2 classification) (*n* = 2669)*. *No pain diagnosis recorded in 37 patients. Abbreviation: NOS = Not Otherwise Specified

The distribution of the primary pain site diagnosis (ICPC-2 classification) is presented in Fig. 2. Low back pain was the most prevalent classification, followed by neck pain and shoulder pain. The 197 physiotherapists treated a median of 18 patients (IQR 13 to 23) each. The median number of treatments was 6 (IQR 3 to 10). Over the whole group, the prevalence of physiotherapy treatments used was exercise therapy 36%, manual therapy 36%, instruction/advice on home exercise 22% and the use of other physical modalities (e.g. electrotherapy and thermotherapy) 6%. At an individual patient level, the treatment recorded over the course of treatment, included exercise therapy at least once for 83% of patients and, when combining exercise therapy and/or instruction/advice on home exercises, the prevalence was 95%.

### Clinical course

On average, patient improvement at the 6-month follow-up was 2.9 (95% Confidence interval CI 2.7 to 3.0) on their pain intensity scores and 9.7 (95% CI 8.9 to 10.5) on their disability scores. The prevalence of being on temporary health-related benefits was reduced from 12% at baseline to 8% at the 6-month follow up - a total reduction of 4.1% (95% CI 3.0% to 5.3%) - when excluding patients who retired or received permanent health-related income support. The percentages of patients perceiving their symptoms as acceptable increased from 32% at 6 weeks to 43% at 3 months and 52% at 6 months – an estimated difference of 19.1% (95% CI 16.3% to 21.7%) On average, patients who perceived their symptoms as acceptable had significantly better pain and disability scores at the three follow up time points than those patients who did not perceive their symptoms as acceptable, mean difference 1.8 (95% CI 1.7 to 2.0) for pain intensity and 7.2 (95% CI 6.4 to 8.0) for disability.

### Predictors of satisfactory outcome

Table 2 shows the results of the final multivariable model, with and without the primary musculoskeletal pain diagnosis variable. Nine predictors were identified: geographic region, health-related income support, compensation claim, duration of pain, frequency of pain, disability level, fear avoidance beliefs, mental health and smoking; with the outcome variable being patients who perceived their symptoms as acceptable during the course of the 6-month follow up. Adding the primary symptom diagnosis to that model had little impact on these associations, as no single pain diagnosis was predictive of a satisfactory outcome. For the selection of variables, changing the significance level to 0.2 added two additional predictors (gender and sleep problems), but with minimal impact on other associations. Similarly, sub-grouping patients with neck and low back pain into those with radiating and non-radiating pain did not affect the results.

**Table 2:** Results of multivariable analysis with rating symptoms acceptable as outcome without (Model 1) and with inclusion of primary pain diagnosis (Model 2)

	Model 1		Model 2	
Variables	OR	95% CI	OR	95% CI
Socio-demographic				
Geographical Regions^a^				
Capital Region of Denmark	1.00		1.00	
Region of Southern Denmark	1.14	0.92–1.41	1.16	0.93–1.43
Central Denmark Region	1.25	1.00–1.57	1.29	1.02–1.62
North Denmark Region	1.41	1.15–1.74	1.44	1.16–1.78
Health-related income support^a^				
None	1.00		1.00	
Temporary	0.73	0.54–1.00	0.77	0.56–1.05
Permanent	0.77	0.54–1.10	0.78	0.55–1.11
Retired	1.18	0.97–1.43	1.22	1.01–1.48
Compensation claim				
No	1.00		1.00	
Yes	0.59	0.40–0.86	0.58	0.40–0.85
Pain and Function				
Pain duration				
< 1 month	1.00			
1–3 months	0.59	0.48–0.73	0.59	0.48–0.72
4–12 months	0.46	0.37–0.58	0.46	0.36–0.58
> 12 months	0.49	0.39–0.60	0.49	0.39–0.60
Disability score 0–100^b^	0.99	0.99–1.00	0.99	0.99–1.00
Frequency of pain (Constant or daily)				
No	1.00			
Yes	0.68	0.54–0.86	0.67	0.53–0.85
Psychological factors				
Fear avoidance 0–30^b^	0.98	0.97–0.99	0.99	0.98–1.00
Mental health score 0–100^b^	1.01	1.00–1.01	1.01	1.00–1.01
Health behaviour				
Smoker				
No	1.00			
Yes	0.87	0.79–0.96	0.87	0.78–0.96
Primary pain diagnosis				
Muscle/Limited function NOS			1.00	
Neck			1.30	0.94–1.80
Upper back/Chest			0.90	0.60–1.34
Low back			1.00	0.73–1.37
Shoulder			0.95	0.66–1.35
Arm/Elbow/Hand			1.04	0.68–1.60
Hip			1.09	0.71–1.67
Knee			0.80	0.53–1.21
Thigh/Lower leg/Foot			0.85	0.58–1.25

^a^ *p*-value < 0.05 for overall test performed with *χ*2 statistics, ^b^ OR per 1 unit increase in scores

*Abbreviations*: *OR* Odds Ratio, *CI* Confidence interval, *NOS* that was Not Otherwise Specified

## Discussion

This study evaluated the clinical course, treatment, prevalence of satisfactory outcome, and predictors of satisfactory outcome in musculoskeletal physiotherapy patients, including the possible influence of a primary pain site diagnosis on those predictors. Over the 6-month follow-up period, statistically significant and clinically relevant improvements were observed across outcomes; however, in keeping with the recurrent and fluctuating nature of many musculoskeletal conditions, only 52% of patients rated their symptoms as acceptable at 6 months. A higher probability of satisfactory outcome was associated with geographic region, being retired, no compensation claim, less frequent pain, shorter duration of pain, lower levels of disability, lower levels of fear avoidance, better mental health and being a non-smoker. Primary pain site diagnosis had little impact on the strength of these associations and did not add any significant predictive ability.

### Methodological considerations

Our study included a large consecutive cohort of patients who were recruited from many physiotherapy practices and therefore the results are likely to reflect primary care musculoskeletal problems as they present to Danish physiotherapists. Information from several data sources was combined (i.e. clinical data, questionnaire data and registry data) allowing the inclusion of a broad variety of variables to describe the clinical course and to identify predictors of outcome.

A limitation of this study is that 33% of the patients invited to participate declined the offer and another 12% had to be excluded due to incomplete data and for other reasons. This modest participation rate may have affected the generalisability of our findings, although it also reflects the general difficulty of recruiting consecutive cohorts of patients from routine clinical practice. Such non-participation rates are common in large population studies and there is evidence from other Danish studies that the estimated associations between variables may not necessarily be biased by non-participation [49, 50]. In our case, missing values in baseline variables were few, but response rates at the three follow-up points were only moderate and there were identified differences between responders and non-responders. Although we conducted analyses using all the available data by use of longitudinal regression modelling, which is robust to missing data [51, 52], this cannot preclude a selection bias. Some would argue that when assessing prognosis, a 12-month follow up would be a preferable endpoint. However, the 6-month endpoint in the current study was chosen as little additional change has been shown to occur beyond this point in common musculoskeletal conditions, such as low back pain [53]. We used an established diagnostic classification system and validated questionnaires; we also chose a main outcome measure that is not commonly used in prognostic studies, albeit that it might provide a more meaningful outcome in patients with musculoskeletal disorders. The concept underpinning the PASS is the level of symptoms beyond which patients consider themselves well (in a satisfactory state) rather than having just experienced a change in symptoms. As this is a state of well-being characterised by at least a state of partial remission, from a patient perspective, it can be considered a highly relevant treatment goal [43, 45, 54]. Understanding factors that are related to achieving and maintaining such a state would provide useful information for daily clinical practice. That significantly better pain and disability scores were observed for patients who perceived their symptoms as acceptable at three follow up points than those patients who did not perceive their symptoms as acceptable, adds to the concurrent validity of this outcome measure.

It could also be argued that diagnostic pain site labelling according to the ICPC-2 system is too broad for musculoskeletal pain conditions, but repeating the analyses after reclassifying neck and low back patients into those with or without radiating pain did not change our results. While no consensus exists on the best method to build models using candidate predictors, some have argued that backward elimination method that we used is the preferred method [48]. Also, while the choice of significance level can effect the number of variables selected, a sensitivity analysis demonstrated that, if the significance level were changed, two additional predictors would have been included (gender and sleep problems) but this would not have substantially altered the results and conclusion of the study. Furthermore, although a large number of candidate variables were included in our study, the achievement of a satisfactory outcome could also be related to other factors that we did not measure, such as patients’ beliefs and expectations of treatment.

### Clinical course

The distribution of gender, age, duration of symptoms and primary pain site diagnoses in our study, is similar to that found in a previous Danish study of patients referred to physiotherapy [5], as well as cohorts of musculoskeletal physiotherapy patients from other countries [55]. The overall improvement on the outcomes of pain intensity, disability and sick leave (temporary health-related income support) was similar in size to those previously observed in musculoskeletal conditions such as low back pain [53] and exceeded a common threshold of clinically relevant important change (i.e. > 30% improvement from baseline) [56]. However, the design of the current study does not allow any judgments to be made about the effectiveness of physiotherapy treatment, as considerable improvement in musculoskeletal pain has been observed without any treatment [57]. Only half of the patients rated their symptoms as acceptable at 6 months, which is in accordance with previous findings in general practice - reporting pain and disability to persist in up to 60% in cohorts of primary care patients with low back, shoulder, and upper extremity pain [58–60]. Thus, despite the physiotherapy treatment in our study, which almost always included active treatment strategies, this relatively moderate success rate suggests either that there is potential for improvement in treatment and/or that musculoskeletal pain conditions are inherently difficult to treat. Contemporary evidence would suggest that shifting from a more traditional physiotherapy pain-centred treatment paradigm to a more function-centred treatment approach focusing on improving function, teaching patients to understand and cope with the episodic nature and fluctuating pattern of musculoskeletal pain, may be key elements to improving the perception of a satisfactory outcome in musculoskeletal physiotherapy patients.

### Predictors of satisfactory outcome

In our study, a number of predictors associated with satisfactory outcome were identified from diverse health domains. Duration of symptoms, disability levels and psychological factors have consistently been found to be associated with subsequent outcomes in multiple prognostic studies in primary care [8, 9, 19]. We found symptoms of shorter duration and less frequent pain to be also associated with a higher probability of satisfactory outcome, consistent with previous studies of different outcomes. The concept of categorising musculoskeletal pain as acute, subacute or chronic by focusing on symptom duration has more recently been challenged by longitudinal studies of trajectories (patterns of change in pain over time) in low back pain [61]. These studies suggest that most people with low back pain have trajectories of either episodic, fluctuating or persistent pain, rather than one well-defined episode. Whether these trajectories can be identified across musculoskeletal pain conditions and thereby improve the prediction of outcome needs further investigation, but approaches that generalise across musculoskeletal conditions would simplify clinical practice. Similar to our findings, being a non-smoker has previously been shown to be associated with a better prognosis among primary care patients seeking care for upper extremity pain [62]. Whereas for other pain sites, similar results have not been reported, partly because smoking has less frequently been included in prognostic studies of musculoskeletal pain [8–12, 19, 63].

Socio-demographic variables that predicted outcome, included compensation claim, geographic region and health-related income support. Having an ongoing compensation claim can be stressful and is a predictor of outcome in secondary care neck and back patients [64, 65]. Although, this involves a very small group among musculoskeletal physiotherapy patients, similar mechanisms could be influential in primary care. The findings that patients were more likely to perceive their symptoms as acceptable in the central and northern geographic regions of Denmark and when retired from the labour market, suggests the prognostic implications of within-country cultural differences and of having no work life demands. Differences in prognostic influences between countries have previously been reported in cohorts of chiropractic spinal pain patients [66], but as far as we are aware, these within-country geographical differences in Denmark among musculoskeletal physiotherapy patients are a novel finding. Whether the higher probability of achieving a satisfactory outcome in more sparsely populated areas (North Region), when compared to densely populated areas (Capital Region), is related to differences in accessibility of physiotherapy services, treatment beliefs, expectations or other factors needs further investigation. Furthermore, the strength of the observed associations was mostly modest and several of the strongest associations were for predictors which are constructs that are not easily modifiable (e.g. duration of symptoms, compensation claims and geographical differences). This currently presents a challenge for clinical practice and prognostic research. Therefore, understanding what potentially modifiable underlying factors may influence such constructs may be an important future research aim [13].

### Influence of primary pain site diagnosis

Primary pain site diagnosis had little impact on the prediction of outcome. The prognosis of patients did not differ across the primary pain site diagnosis from that of patients who were classified with non-specific muscular symptoms and limited function. These results are supported by those from cohort studies in the USA, showing anatomical pain sites did not influence the predictive value of psychological factors, such as fear avoidance beliefs and depressive symptoms, in patients seeking physiotherapy treatment for musculoskeletal pain [67, 68]. Traditionally, clinical practice prognosis and treatment has been based on medical diagnoses but the central role of diagnosis has recently been challenged in conditions such as musculoskeletal pain, where other factors have been shown to be more influential on the outcome [6]. In patients with musculoskeletal pain, many symptoms (such as pain, stiffness, and pain interference with work and daily routine) are common regardless of where the primary pain site is. In musculoskeletal pain conditions, prognosis seems to be less about ‘where you have it’ and more about ‘how much pain and disability you have and your pain perceptions’. Diagnosis is likely to be most relevant in conditions where there is an available treatment that effectively targets the causal pathways of a specific disease. In health conditions such as musculoskeletal pain, physiotherapy treatment strategies largely depend on the same strategies, such as advice, reassurance, manual therapy and exercise, and therefore are probably better understood and managed within a prognostic framework.

## Conclusion

Among Danish musculoskeletal physiotherapy patients, only one in two perceived their symptoms as acceptable at 6 months, despite the average improvement being above the threshold for clinically important change. A number of predictors of satisfactory outcome were identified, which appear to be prognostic regardless of primary pain site diagnosis and which may have a role in generic prediction models for physiotherapy patients with musculoskeletal pain. While clearly there is a role for studies of single-site musculoskeletal pain, our results also support the need for more research that offers insights into the drivers of recovery regardless of pain site and provides generic tools to assist the management of musculoskeletal pain, whether it be single-site or multi-site.

## Additional file

Additional file 1: Cross-cultural adaptation and validation of the Danish version of the Standard Evaluation Questionnaire (SEQ) modules. (DOCX 251 kb)

## Abbreviations

GEE: Generalised Estimating Equation
ICPC-2: International Classification for Primary Care 2^nd^ edition
MH-5: Mental Health Scale – Five
MSPQ: Musculoskeletal Pain Screening Questionnaire
NOS: Not Otherwise Specified
PASS: Patient Acceptable Symptom State
SEQ: Standard Evaluation Questionnaire (SEQ)
