# Supplementary material for: Clinical course and prognosis of musculoskeletal pain in patients referred for physiotherapy: does pain site matter?
Source: BMC Musculoskelet Disord. 2017 Mar 29;18:130. doi: 10.1186/s12891-017-1487-3 (PMC5371202; doi:10.1186/s12891-017-1487-3)
Supplement: Additional file 1: — Cross-cultural adaptation and validation of the Danish version of the Standard Evaluation Questionnaire (SEQ) modules. (DOCX 251 kb) [file 12891_2017_1487_MOESM1_ESM.docx]

**Additional file 1**

**Cross- cultural adaptation and validation of the Danish version of the Standard Evaluation Questionnaire (SEQ) modules**

## Methods

The SEQ-pain, SEQ-disability and SEQ-sleep models were translated from Swiss German into Danish and pilot tested in 7 physiotherapy patients and adapted to the Danish culture. The measurement properties of the Danish version of the SEQ were evaluated in a test-retest design in a separate patient sample (n=68, 52% females, mean age 47) recruited in one physiotherapy practice using the same inclusion criteria as in the main study. Three validated-region specific scales were also completed according to their primary complaint, so that the concurrent validity of the SEQ could be evaluated: the Disabilities of the Arm, Shoulder and Hand questionnaire (DASH), Oswestry Disability Index (ODI) and Rheumatoid and Arthritis Outcome Score (RAOS).

The sample size for the test-retest study of the SEQ modules was based on the international recommendation of including at least 50 participants for assessing measurement properties. Reliability and internal consistency was assessed by calculation of Inter Class Correlation coefficients (ICC_2.1_), Kappa and weighted Kappa with squared weights (ΚW^2^) and Cronbach’s α statistics. Reliability statistics can range from 0.0 to 1.0, where a high value (close to 1.0) implies high reliability and a low value (close to 0.0) implies low reliability. Systematic bias between test and retest ratings was determined by paired t-tests of sum scores and McNearmar Tests of single items. Convergent construct validity was evaluated by correlation analyses (Spearman’s rho) between SEQ-pain, SEQ-disability and SEQ-sleep sum scores and scores of the DASH, the ODI and the RAOS.

## Results

The forward and back translations did not reveal any linguistic difficulties and the pilot study showed that all patients found the SEQ easy to complete and relevant to their symptoms. In the reproducibility component, a total of 63 patients (93%) completed both the first and second questionnaire, with a median of 5 days (interquartile range 3 to 6) between administrations. ICC for the SEQ-pain module ranged from 0.57 to 0.85 (only one coefficient <0.60) for the intensity of pain in different body regions, with the median Kappa value for location of pain being 0.84 (range: 0.67 to 0.90) and 0.94 for the number of pain sites. For the single categorical items, the Kappa (ΚW^2^) was 0.84 for questions about the duration of pain, 0.68 for the frequency of pain, and 0.95 for the use of pain medication. The ICC was 0.90 for the sum score on pain during activity, with Cronbach’s α being 0.91. For the SEQ-disability sum score ICC and Cronbach’s α coefficients were both 0.92 and ICC for SEQ-sleep sum score was 0.82. Moderate to strong correlations (spearman’s rho >0.50) were found between the condition-specific scales (DASH, ODI and RAOS) and SEQ-pain, SEQ-disability and SEQ-sleep sum scores. No systematic bias was observed between test and retest sum scores or single items.

**Conclusion**

The reliability of the Danish version of the SEQ was found to be acceptable and convergent construct validity was confirmed. The SEQ is a reliable tool for assessing pain, disability and sleep disturbance in Danish-speaking physiotherapy patients with various musculoskeletal disorders.

## Danish version of (SEQ) modules
